# Interactions of *Candida albicans* with host epithelial surfaces

**DOI:** 10.3402/jom.v5i0.22434

**Published:** 2013-10-21

**Authors:** David W. Williams, Rachael P. C. Jordan, Xiao-Qing Wei, Carlos T. Alves, Matt P. Wise, Melanie J. Wilson, Michael A. O. Lewis

**Affiliations:** 1 Tissue Engineering and Reparative Dentistry, School of Dentistry, College of Biomedical and Life Sciences, Cardiff University, Cardiff, UK; 2 IBB–Institute for Biotechnology and Bioengineering, Centre of Biological Engineering, University of Minho, Braga, Portugal; 3 Adult Critical Care, University Hospital of Wales, Cardiff, UK

**Keywords:** oral microbiology, biofilm, virulence factors, pathogenesis

## Abstract

*Candida albicans* is an opportunistic, fungal pathogen of humans that frequently causes superficial infections of oral and vaginal mucosal surfaces of debilitated and susceptible individuals. The organism is however, commonly encountered as a commensal in healthy individuals where it is a component of the normal microflora. The key determinant in the type of relationship that *Candida* has with its host is how it interacts with the epithelial surface it colonises. A delicate balance clearly exists between the potentially damaging effects of *Candida* virulence factors and the nature of the immune response elicited by the host. Frequently, it is changes in host factors that lead to *Candida* seemingly changing from a commensal to pathogenic existence. However, given the often reported heterogeneity in morphological and biochemical factors that exist between *Candida* species and indeed strains of *C. albicans*, it may also be the fact that colonising strains differ in the way they exploit resources to allow persistence at mucosal surfaces and as a consequence this too may affect the way *Candida* interacts with epithelial cells. The aim of this review is to provide an overview of some of the possible interactions that may occur between *C. albicans* and host epithelial surfaces that may in turn dictate whether *Candida* removal, its commensal persistence or infection follows.

The *Candida* genus consists of approximately 200 species of ‘yeast-like’ fungi and collectively represents a highly heterogenic group (1). Taxonomically, the *Candida* genus is in the class Deuteromycetes, and a feature of *Candida* species is their ability to grow polymorphically, either in the form of budding yeasts (blastoconidia) or filaments (true hyphae and pseudohyphae). The reason for this heterogeneity in the *Candida* genus largely stems from the fact that historically, designation of organisms to the genus was based on the absence of a known sexual reproduction stage. In many instances, *Candida* species have since been shown to reproduce sexually, but have retained their taxonomic status within *Candida*. As a consequence, *Candida* species can differ greatly in terms of their biochemistry, morphology, genetic composition and, importantly, their ability to instigate human infection.

In the case of human infections caused by *Candida*, the terms candidosis (sing.) or candidiasis are used, and candidoses (pl.) can broadly be categorised as being systemic or superficial. Systemic infections generally develop in severely immunocompromised individuals and whilst these infections are relatively rare, they are associated with high mortality. In contrast, superficial infections on moist mucosal surfaces, such as those of the mouth and vagina are more prevalent, but have less damaging effects to the host.

Approximately, 20 *Candida* species have, at some point, been associated with causing candidosis in humans. The species most frequently isolated from humans and the causative agent of the majority of infections is, however, *Candida albicans* and it is this species that is the focus of this review.

*Candida albicans* is an opportunistic pathogen and generally exists as a harmless commensal of humans, primarily on moist mucosal surfaces, particularly of the gut, vagina, and oral cavity. Depending on the population studied, commensal carriage in the oral cavity can range between 40 and 60% (2). In the case of the vagina, *Candida* colonisation rates again vary with studied groups, with carriage rates of 41 and 21% reportedly occurring in type 1 and type 2 diabetic patients, respectively (3). Women who are pregnant also reportedly have a high incidence of vaginal carriage (4), and vaginal candidosis is one of the most common superficial infections in reproductive-age women (5).

Given that *C*. *albicans* colonises host surfaces at such a high prevalence, infections are unsurprisingly often endogenous (6), occurring when there is an ecological shift in the microbiological community, frequently due to debilitation in the host's immune system. Receipt of a broad-spectrum antibiotic, a high frequency intake of carbohydrates, hormonal imbalances, and poor nutrition may also be contributory factors. Interestingly, in the case of oral candidosis four clinically distinct forms of infection are recognised (Fig. 1) and these could reflect different forms of interaction between the colonising *Candida* and host epithelium. The four clinically distinct primary forms of oral candidosis are acute erythematous candidosis, pseudomembranous candidosis, chronic erythematous candidosis, and chronic hyperplastic candidosis. Clinical symptoms of acute erythematous candidosis include redness and soreness of the oral mucosa with the tongue most often affected. Pseudomembranous candidosis is most common in infants and immunocompromised people and typically manifests as creamy white plaques or patches on oral tissues that can usually be scraped off. Chronic erythematous candidosis presents as localised erythema in regions of ill-fitting or inadequately cleaned dentures. Chronic hyperplastic candidosis is seen as firmly adhered white patches on the oral mucosa.

**Fig. 1:**
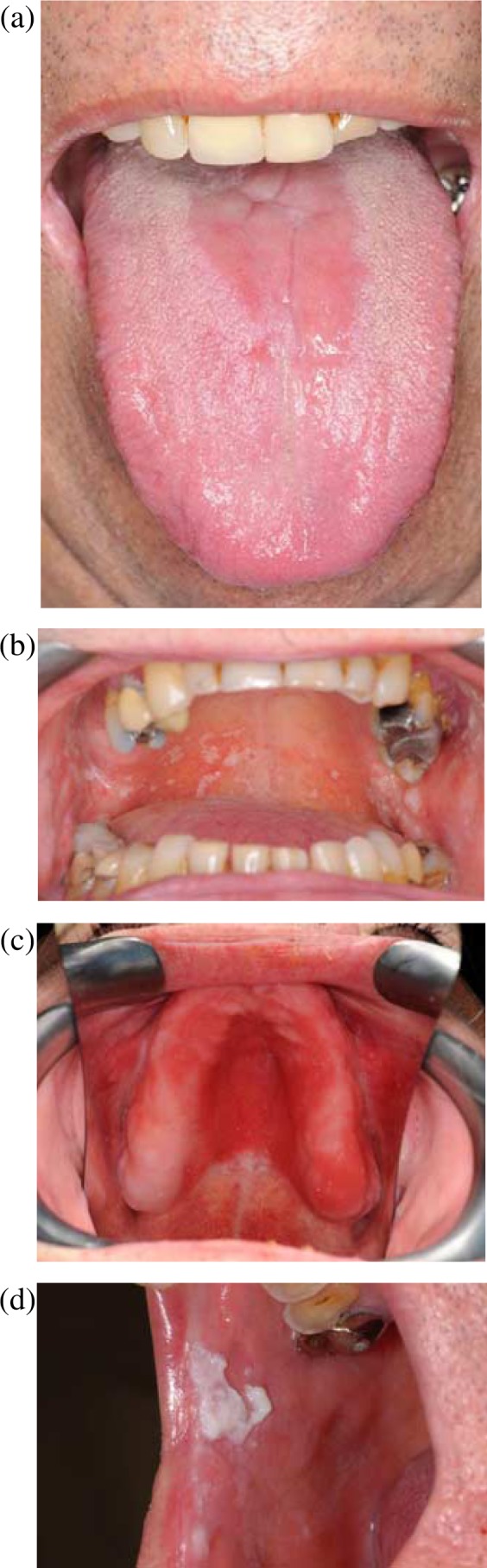
Clinically distinct forms of primary oral candidosis. (a) Acute erythematous candidosis; (b) pseudomembranous candidosis; (c) chronic erythematous candidosis; (d) chronic hyperplastic candidosis.

To successfully persist within the host environment, either as a commensal or as a pathogen, *Candida* first has to adhere and then colonise host surfaces. These surfaces may take the form of the biomaterials of medical devices, for example, the acrylic of a denture, or the host's mucosal surfaces.

## Adherence of *Candida* to mucosal surfaces

The process of initial adherence of *Candida* to human epithelial surfaces is complex and multifactorial. Cell surfaces (both *Candida* and epithelial cells) are generally negatively charged, and establishing successful adherence is, in part, dependent on the sum of non-specific factors contributing to the total free energy of interaction. These include attractive Lifshitz–van der Waals forces, hydrophobic interaction, and Brownian movement forces, as well as the repulsive effects of the electrical double layer of cells. Such interactions form the basis of the extended Derjaguin–Landau–Verwey–Overbeek (DLVO) theory (7).

Once the ‘long-distance’ repulsive forces have been overcome, adherence of *Candida* is then mediated by specific molecules, referred to as adhesins, on the fungal cell surface and these interact with specific ligands on the host cell surface (Table 1). Adhesins on the cell surface of *C. albicans* can interact with serum proteins, components of the extracellular matrix (ECM), immobilised ligands such as cadherins or integrins, or indirectly via other microorganisms (28). An important serum component that *C. albicans* can bind to is Factor H (FH; 29) which is a key regulator of the alternative pathway (AP) of complement and incorporation of FH on the surface of *C. albicans* prevents AP activation (30). Laminin, fibronectin, collagen, entactin, vitronectin, and tenascin are all ECM proteins that *C. albicans* can interact with (31).

**Table 1:** Examples of *Candida albicans* adhesins and associated host cell ligands

*Candida* adhesin	Host cell receptor	References
Integrin analog (INT)	iC3b, Arginine–glycine–aspartic acid (RGD)	(8– 11)
Fibronectin adhesin (FN)	Fibronectin and vitronectin receptors	(12–14)
Fucoside-binding adhesin	Glycoside (glycoprotein or glycolipid) receptor	(15–18)
GlcNAc-binding protein	N-Acetylglucosamine	(15–17)
Fimbrial adhesin	βGalNAc(1–4β-Gal)	(19)
Hyphal wall protein 1 (HWP1)	A substrate of epithelial cell-associated transglutaminases facilitating cross-linking with epithelial cells	(20, 21)
Agglutinin-like sequence (ALS) family	Multiple receptors including E-cadherin, N-cadherin and host cell ferritin	(22–25)
Enhanced adherence to polystyrene (*EAP1*)	Host cell targets not yet identified	(26–27)

Members of the agglutinin-like sequence (ALS) gene family of *C. albicans* encode for large cell-wall glycoproteins, some of which are implicated in the adhesion of the organism to host surfaces (25, 32). The ALS gene family comprises of eight members (Als1–Als7, and Als9) and all have a similar three-domain structure and are associated with the β-1,6-glucan of the cell wall of *C. albicans* (23). In the case of *C. albicans*, Als3 appears to play a key role in adhesion to oral epithelial cells, and it is also related to the extent of subsequent epithelial damage and induction of epithelial cytokines (33).

Hyphal wall protein 1 (Hwp1; encoded for by the *HWP1* gene) is another protein involved in *C. albicans* adhesion to epithelial cells and this protein is perhaps the most widely studied adhesin of *C. albicans* (34). Glutamine residues in the N-terminal domain of Hwp1 can be cross-linked to unidentified host proteins by host transglutaminase activity and this leads to covalent attachment of the yeast to host epithelial cells. This interaction has been shown to be important for *C. albicans* colonisation within the oral cavity (35).

The β-1,3-glucan motif of the cell wall of *C. albicans* and indeed other pathogenic fungi (36), has been shown to interact with Dectin-1 on the surfaces of host cells, primarily on phagocytotic cells including dendritic cells within the oral epithelium. As such, several studies have shown that Dectin-1 belongs to the armoury of pathogen recognition molecules participating in host defence against fungal pathogens, including *Candida* species and *Aspergillus* species (37, 38). Dectin-1 can synergise with toll-like receptor (TLR) 2 and TLR4 signals and promote Th1 and Th17 responses to activate antifungal host defences (39–41). Further detail concerning Dectin-1 and fungal interactions in respect to immune responses is provided later in this review.

Recently, the gene encoding the C. albicans protein, EAP1 (Enhanced Adherence to Polystyrene) was identified. This gene was originally investigated because of its ability to encode for a protein mediating adhesion to polystyrene of a *Saccharomyces cerevisiae* flocculin-deficient strain. *EAP1* encodes for a glycosylphosphatidylinositol-anchored, glucan-cross-linked cell-wall protein that has since been shown to facilitate adhesion of *C. albicans* to epithelial cells as well as polystyrene (28).

Once adherence to mucosal surfaces has been established, colonisation and growth of *C. albicans* is required to maintain the presence of the organism at the host site. The extent of this colonisation is key to determining whether eradication, commensal carriage, or infection subsequently follows. The ability of *C. albicans* to generate a biofilm on host surfaces, including mucosa, is also an important attribute toward such persistence.

## Biofilm formation by *C. albicans* on mucosal surfaces

**Fig. 2:**
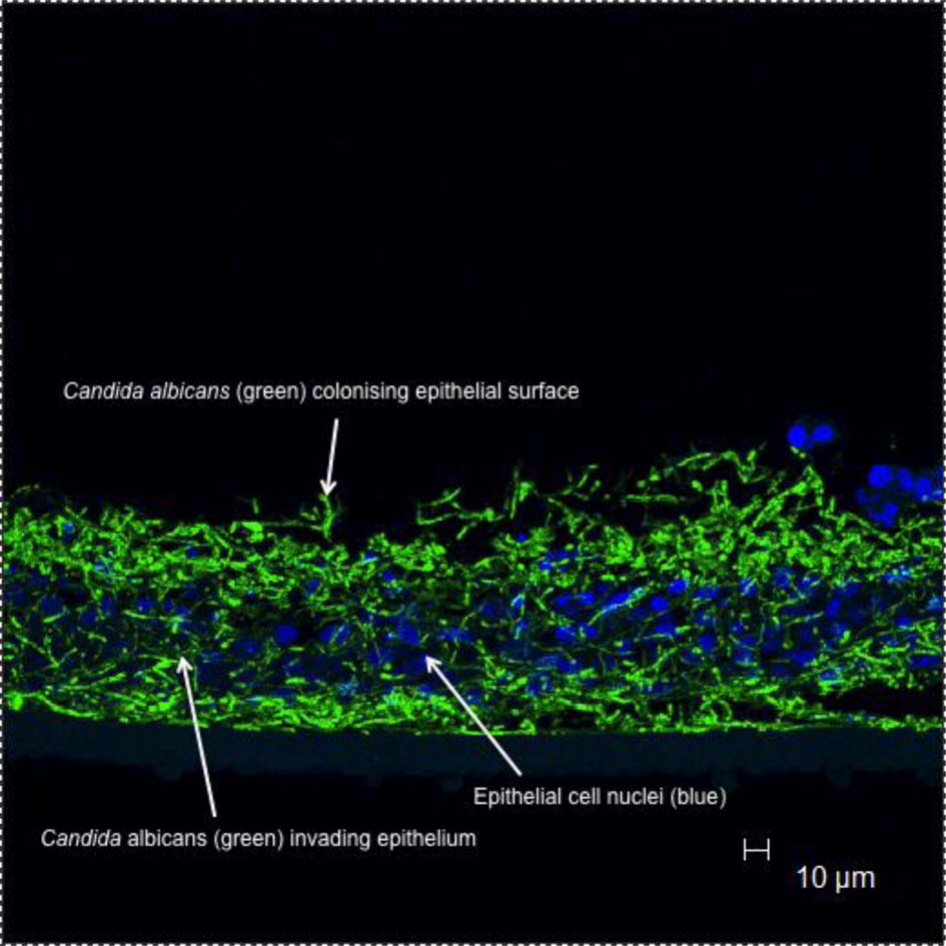
*Candida albicans* biofilm formation on oral mucosal surfaces. Arrows indicate green fluorescing *C. albicans* (stained with a labelled peptide nucleic acid probe) infecting a reconstituted oral epithelium generated commercially from transformed human keratinocytes of the cell line TR146 (from a squamous cell carcinoma of the buccal mucosa; SkinEthic Laboratories, Nice, France); nuclei of epithelial keratinocytes are shown as blue (Hoescht staining).

Biofilms are defined as microbial communities that are often attached to solid substrata with the biofilm cells themselves embedded within extracellular polymeric substances (EPS) that they have generated. *C. albicans* is particularly adept at forming biofilms on the acrylic of dentures and also on mucosal surfaces (Fig. 2). In the case of pseudomembranous candidosis, the pseudomembranes that develop on the oral mucosa have been shown to be typical biofilms and linked to the recalcitrant nature of the condition (42). Once the biofilm has formed, the EPS encasing the cells may contribute to persistence of the organism by several possible mechanisms. First, the EPS may serve to sequester antimicrobial substances that are present in oral secretions or within administered agents thereby limiting diffusion into the biofilm and access to cells. Similarly, restricted access of phagocytotic cells to *C. albicans* within the biofilm will also occur. It has also been suggested that altered cell phenotypes, potentially with reduced growth rates of biofilm cells provides an additional means of protection against host defence molecules. An important regulator of *C. albicans* biofilm formation is the transcription factor Bcr1, which is a positive regulator of several candidal adhesin genes including *HYR1*, *HWP1*, *CHT2*, *ECE1*, *RBT5*, *ALS1*, and *ALS3* (43, 44). The importance of Bcr1 in *C. albicans* biofilm formation within in a mouse model of oral infection has recently been demonstrated (45).

## Damage induced by *C. albicans* to epithelial cell surfaces

To propagate at mucosal sites, nutrients may be acquired from the surrounding milieu or through degradation of host tissue structures. The latter may also allow penetration of *C. albicans* hyphae into deeper layers of the epithelium (Fig. 2), which would further enhance persistence of the organism on oral surfaces, which, in the oral cavity has a high cellular turnover partly serving as a defence mechanism to remove colonised cells.

As previously mentioned, *C. albicans* is an opportunistic pathogen and as such it can be argued that it does not possess potent virulence factors, certainly when compared with other strict pathogens. However, *C. albicans* can generate a number of hydrolytic enzymes with broad substrate activity that can damage host cell structures. Perhaps, the most extensively studied extracellular hydrolytic enzymes of *C. albicans* are the secreted aspartyl proteinases (SAPs).

The SAP family of *C. albicans* is currently known to comprise 10 genes encoding for proteinases with masses of 35–50 kDa. SAPs 1–3 and SAPs 4–6 are thought to represent two subfamilies (46). The SAP genes are differentially regulated depending on the surrounding environment and are thought to be involved in the pathogenesis of *C. albicans*. *SAP1*–*6* gene expression appears to be related to adherence, tissue damage, and changes in the immune response (46–49). SAPs 4–6 are expressed by *C. albicans* during hyphal invasion of a reconstituted human oral epithelium (RHE; 50) and oral infection (51). SAPs 4–6 are also linked with hyphal formation, invasion of the epithelium (52), and apoptosis of epithelial cells (53). SAPs 2 and 6 are also potent inducers of IL-1β, TNF-α, and IL-6 production by monocytes (54). The SAP gene products are suggested to contribute to various virulence processes *in vitro* including adherence to and invasion of the epithelial cells (55, 56). However, all the SAPs have distinct pH optima and the extent of their functional activity at the generally neutral pH of oral mucosa remains to be ascertained.

E-cadherin is a protein associated with epithelial cell junctions that serves to maintain a functional barrier to invasion. *In vitro* breakdown of E-cadherin, produced by oral epithelial cells by SAP5, has been demonstrated and this could represent a mechanism by which *C. albicans* mediates invasion of oral mucosa (56).

In addition to SAPs, *C. albicans* also has two other gene families, namely the lipases (LIP) and phospholipases (PL) that produce extracellular hydrolytic enzymes that could play roles in candidal adhesion, nutrient acquisition and invasion of epithelial surfaces (57, 58). The LIP gene family of *C. albicans* comprises at least 10 genes (LIP1–10) (59), whilst seven phospholipase genes of *C. albicans* have been identified (PLA, PLB1, PLB2, PLC1, PLC2, PLC3, and PLD1) (60). Constitutive expression of the LIP genes and PLB has been demonstrated in *C. albicans* biofilms generated on an RHE (61).

## Epithelial cell responses to *C. albicans*

Epithelial cells of host mucosal surfaces represent the first line of defence against *Candida* infection. As key cells in the innate immunity of the host, epithelial cells express pattern recognition receptors (PRRs), which recognise *C. albicans*. PRRs interact with pathogen-associated molecular patterns (PAMPs) on microbial cells and examples of these in *C. albicans* include cell-wall components and nucleic acids. PRRs are divided into three major groups, TLRs, C-type lectin receptors (CLRs) and nod-like receptors (NLRs). Within these receptor groups, only certain TLRs and CLRs on epithelial surfaces recognise *Candida*. In addition to PRRs, other cell-surface proteins, such as E-cadherin and Epidermal Growth Factor Receptor (EGFR), can also recognise *Candida* and as discussed previously, these are unsurprisingly implicated in *Candida* adherence and endocytosis (62, 63).

Cell-surface recognition of *Candida* produces a cascade cell signaling reaction, which leads to gene expression in epithelial cells for a number of growth factors, chemokines/cytokines, antimicrobial peptides, and cell matrix proteins (64, 65). Epithelial responses to *Candida* may not however, result in a strong host immune response and inflammation. Indeed, certain candidal factors as well as proteins produced by epithelial cells may actually result in anti-inflammatory effects and subsequent immune tolerance (66). The precise features that determine whether epithelial cells induce inflammation or are acquiescent toward *C. albicans* remain unclear. In the following section, we will summarise the general mechanisms involved in epithelial cell responses to *Candida*, including cell-surface receptor binding, cell signaling triggering, and the factors produced by the epithelial cells.

TLRs are a family of PRRs whose involvement in host innate immune responses to various pathogens has been well studied. Up to 13 TLRs have been identified in both humans and mice. Expression of TLR1, 2, 4, 5, and 6 has been demonstrated in human mucosal epithelial cells (64–68), and their expression in response to *C. albicans* infecting both oral and vaginal epithelial cell lines has been shown to be similar (69). The exact composition of the PRRs used by epithelial cells in response to infections with *C. albicans* is, however, unknown (69). TLR2 and TLR5 are both expressed at high levels by oral cells and are frequently associated with epithelial repair, growth, and survival (70, 71).

*Candida* stimulates human epithelial cells to express (granulocyte-macrophage colony-stimulating factor) GM-CSF, which is a highly potent cytokine that stimulates dendritic cell maturation to mediate mucosal inflammation. Interestingly, TLR4 is not involved in GM-CSF stimulation and it has also been shown that candidal viability is also required in GM-CSF induction (72).

Aside from TLRs, perhaps the most important PRRs for *Candida* recognition are CLRs. CLRs comprise a family of six cell-surface proteins. Dectin-1 and Dectin-2 belong to this family, and are confirmed receptors for *Candida* recognition (73–75). Whilst the role of Dectin-1 and 2 in host immunity against *Candida* infection has been extensively studied in animal models and human macrophages/dendritic cells, only Dectin-1 expression in human oral gingival epithelial cells has been reported, and its expression is at best weak (76). Therefore, the exact role of Dectin-1 expression in oral mucosal immunity remains unclear.

Epithelial cells can also recognise the morphology of the colonising *Candida*. *C. albicans* yeast and hyphae both trigger Nuclear Factor Kappa β (NFkβ) activation in epithelial cells, but NFkβ activation alone does not lead to cytokine release. Only *C. albicans* hyphae appear to be able to also induce mitogen-activated protein kinases (MAPK) phosphorylation, which combined with NFkβ activation results in production of IL-6 and GM-CSF by epithelial cells (34, 77, 78). Despite the identification of such downstream inflammatory signaling cascades, the oral epithelial receptor(s) that induce(s) cytokine responses to *Candida* have yet to be identified. The receptors described above might be involved in differential detection of *Candida* hyphae, thus representing a possible mechanism by which the host distinguishes between commensal *Candida* yeast carriage (resulting in immune tolerance) and invasive *Candida* hyphal infection (resulting in inflammatory immune responses).

Many downstream mechanisms have been identified as influencing immune tolerance and activation following *Candida* colonisation. Examples include involvement of the resident macrophages in the mucosa that produce anti-inflammatory cytokines to regulate host immune responses (66). Nevertheless, details of the mechanisms of *Candida* recognition and host tolerance by mucosal epithelial cells still need to be clarified.

*Candida* also induces *in vitro* upregulation of various antimicrobial peptides such as β-defensins and LL-37 (79, 80), which are known to have candidacidal activity and could play significant roles in combating infections and invasion, as well as initiating other immune responses (81, 82).

## Summary

It is clear that a delicate balance exists between *C. albicans* and host epithelial surfaces. The type of response elicited by the epithelial surface to colonising *Candida* is extremely important given that such surfaces are the first line of defence of the host to infection. The nature of mucosal responses is affected by many variables including host factors such as immune dysfunction, underlying disease, other forms of host debilitation, and the composition of the existing microflora community. In addition, there are factors associated with the strain of *C. albicans* involved that are also important in determining responses of the epithelium. These include the level of expression of putative virulence factors including cell-surface adhesins, extracellular hydrolytic enzymes, and the type of morphology exhibited by the colonising *C. albicans*. Given the heterogeneity associated with such factors in both the *Candida* genus and amongst strains of *C. albicans*, it could readily be postulated that those strains able to adapt to the conditions at the mucosal surface without inducing host responses represent those most likely to successfully persist as commensals. Strains that rely on virulence factors to persist are those that lead either to pathology or become eradicated through the activity of host defences.
